# Retraction Note: miR-29c-3p regulates proliferation and migration in ovarian cancer by targeting KIF4A

**DOI:** 10.1186/s12957-025-03994-w

**Published:** 2025-08-28

**Authors:** Songwei Feng, Shanhui Luo, Chenchen Ji, Jia Shi

**Affiliations:** 1 Department of Gynecology, The Second Affiliated Hospital of Soochow University, Suzhou, People’s Republic of China; 2 Orthopedic Institute, Soochow University, Suzhou, People’s Republic of China; 3 Department of Laboratory, The Affiliated Wuxi Maternity and Child Health Care Hospital of Nanjing Medical University, 48 Huaishuxiang, Wuxi, Jiangsu Province 214002 People’s Republic of China

**Retraction Note: World J Surg Oncol 18, 315 (2020)**

**https://doi.org/10.1186/s12957-020-02088-z**

The Editor-in-Chief has retracted this article. After publication, concerns were raised regarding Fig. 2E, specifically:

- The shKIF4A image appears highly similar to Fig. 2B MDA-MB-321 200 nM image in [1] and Fig. 6E shTRL7 in [2];
- The sh-NC image appears highly similar to Fig. 2B MDA-MB-321 100 nM image in [1].

The authors have been unable to provide the raw data upon request. The Editor-in-Chief therefore no longer has confidence in the presented data.

The authors disagree with this retraction.
